# Attitudes to specialist palliative care and advance care planning in people with COPD: a multi-national survey of palliative and respiratory medicine specialists

**DOI:** 10.1186/s12904-018-0371-8

**Published:** 2018-10-15

**Authors:** Natasha Smallwood, David Currow, Sara Booth, Anna Spathis, Louis Irving, Jennifer Philip

**Affiliations:** 1 Department of Respiratory and Sleep Medicine, The Royal Melbourne Hospital, Melbourne, VIC 3050 Australia; 2 Department of Medicine (Royal Melbourne Hospital), University of Melbourne, Melbourne, VIC 3050 Australia; 3 IMPACCT - Improving Palliative, Aged and Chronic Care through Clinical Research and Translation, Faculty of Health, University of Technology Sydney, Ultimo, NSW Australia; 4 University of Cambridge, Cambridge, UK; 5 Cambridge University Hospitals NHS Foundation Trust, Cambridge, UK; 6 Palliative Medicine, St Vincent’s Hospital and Victorian Comprehensive Cancer Centre, University of Melbourne, Melbourne, Australia; 7 St Vincent’s Hospital, Victoria Parade, Melbourne, VIC 3065 Australia

**Keywords:** COPD, Survey, Health professionals, Palliative care, Attitudes, Advance care planning

## Abstract

**Background:**

Chronic obstructive pulmonary disease (COPD) guidelines recommend early access to palliative care together with optimal, disease-directed therapy for people with advanced disease, however, this occurs infrequently. This study explored the approaches of respiratory and palliative medicine specialists to palliative care and advance care planning (ACP) in advanced COPD.

**Methods:**

An online survey was emailed to all specialists and trainees in respiratory medicine in Australia and New Zealand (ANZ), and to all palliative medicine specialists and trainees in ANZ and the United Kingdom.

**Results:**

Five hundred seventy-seven (33.1%) responses were received, with 440 (25.2%) complete questionnaires included from 177 respiratory and 263 palliative medicine doctors. Most respiratory doctors (140, 80.9%) were very or quite comfortable providing a palliative approach themselves to people with COPD. 113 (63.8%) respiratory doctors recommended referring people with advanced COPD to specialist palliative care, mainly for access to: psychosocial and spiritual care (105, 59.3%), carer support (104, 58.5%), and end-of-life care (94, 53.1%). 432 (98.2%) participants recommended initiating ACP discussions. Palliative medicine doctors were more likely to recommend discussing: what palliative care is (*p* < 0.0001), what death and dying might be like (p < 0.0001) and prognosis (*p* = 0.004). Themes highlighted in open responses included: inadequate, fragmented models of care, with limited collaboration or support from palliative care services.

**Conclusions:**

While both specialties recognised the significant palliative care and ACP needs of people with advanced COPD, in reality few patients access these elements of care. Formal collaboration and bi-directional support between respiratory and palliative medicine, are required to address these unmet needs.

## Background

Chronic obstructive pulmonary disease (COPD) is characterised by airflow limitation, persistent symptoms and respiratory failure [1]. By 2020, COPD is projected to be the third leading cause of death worldwide [2]. Yet despite many people expressing a desire to die at home, seven out of ten people with COPD die in hospital in Australia and the United Kingdom (UK) [3, 4].

Many patients with severe COPD experience reduced quality of life due to severe, chronic breathlessness, which persists at rest or on minimal exertion despite optimal treatment of the underlying causes [5–7]. Disease progression is also reflected in reduced function, limiting basic activities of daily living and requiring additional support from informal and paid caregivers, often for long periods of time [8]. In addition to other physical symptoms such as cough, pain, anorexia and fatigue, many patients have significant psychological comorbidity, with all these factors contributing to reduced quality of life and increasing social isolation for both patients and caregivers [9, 10].

Given the significant physical, psychosocial and communication needs of people with COPD [11], guidelines recommend that patients with advanced disease should receive early access to palliative care in conjunction with optimal, disease-directed therapy [1, 12]. Therefore all health professionals have a role in providing palliative care to their patients [12]. Indeed when palliative care is provided by the usual treating clinician (such as the general practitioner or a medical specialist), it is termed *generalist* palliative care or a *palliative approach* [13]. By contrast, *specialist* palliative care is provided by health professionals who have specialist qualifications and/or significant experience in palliative care. Specialist palliative care teams not only support the usual treating clinician to offer a palliative approach, but have an important role in caring for people with challenging symptoms or complex needs [12]. Thus a palliative approach and specialist palliative care are not separate entities, but may be complementary aspects of care for patients with advanced COPD.

Despite guidelines recommending palliative care for people with advanced COPD, referral to specialist palliative care service occurs infrequently [14–16]. Only 1.7% of patients with end-stage COPD in the USA were referred to specialist palliative care when admitted with an exacerbation [16]. Similarly, in the UK and Australia only 16.7–17.9% of COPD patients accessed any specialist palliative care in their last year of life [15, 17, 18]. Yet people with severe COPD have documented needs which are similar to those of people at the end-of-life, irrespective of the underlying life-limiting illness [19]. Advance care planning (ACP), which by definition should include discussion of each patient’s palliative care wishes, also seldom occurs in routine practice [20–22].

Well-described barriers to accessing palliative care include difficulty prognosticating in COPD due to the variable disease trajectory [23, 24], clinicians lacking time to discuss palliative care or being fearful of taking away hope [25], and availability of specialist palliative care from services which are already overburdened [26, 27]. Given these issues, a survey was undertaken to explore physicians’ knowledge and practices managing people with advanced COPD. This manuscript reports current beliefs and practices of respiratory and palliative medicine doctors regarding the role of specialist palliative care and advance care planning in patients with severe COPD.

## Methods

The full methodology including the research questionnaire utilised for this study have been published elsewhere [28], therefore the methods are summarised here. A voluntary survey was designed for specialists and specialist trainees working in respiratory medicine in Australia and New Zealand (ANZ) and palliative medicine in ANZ and the United Kingdom (UK). The questionnaire included a case vignette describing an outpatient with severe COPD, receiving maximum disease modifying therapies and worsening, severe chronic breathlessness (modified Medical Research Council breathlessness score of 4). Survey participants were told the case patient did not have anxiety and was *not* in the terminal phase (last few days) of his illness. Respondents were asked to consider how they would manage the case patient or people with COPD similar to the case. All survey questions (including demographic information at the start of the survey) required an answer, before the respondent could proceed to the next question.

The link to the online survey (written in Survey Monkey) was distributed by the Thoracic Society of Australia and New Zealand, the Australian and New Zealand Society of Palliative Medicine, and the Association for Palliative Medicine of Great Britain and Ireland to their members. Each society member received two email invitations including the survey link, 2–4 weeks apart. The online survey was open to participants for six months from August 2015 to February 2016, with participants only able to submit one response from their IP address. Consent to participate was implied by completion of the survey questionnaire. Ethics approval was granted by the Melbourne Health Research Office (QA2014171). Separate ethics approvals from each specialist society were not required. Similarly health professional research studies that did not collect sensitive data, did not require ethics approval in the UK in 2015 [29].

### Statistical analysis

Demographic data and responses are reported descriptively using frequencies and proportions. The Pearson Chi-Square test was used to identify associations between participants’ responses and exposure variables measured as proportions (age, gender, country, specialty, position and location of practice); and Student’s t test was used for exposures measured as continuous numerical values (mean years worked in specialty and mean number of patients with severe COPD seen per month). Statistical analyses were performed using IBM SPSS Statistics Version 24.0, with a *p*-value of less than 0.05 indicating statistical significance. Free text responses to open ended questions were analysed using thematic analysis.

## Results

Five hundred and seventy-seven (33.0%) responses were received from 1047 doctors working in palliative medicine (323 in ANZ and 724 in the UK) and 702 working in respiratory medicine who were emailed the survey link. Responses were excluded from participants who: provided incomplete responses regarding patient management (94), were not respiratory or palliative medicine doctors (35), or were non-medical (8). Of the 440 (25.2%) responses included, 263 (25.1%) were palliative medicine doctors and 177 (25.2%) participants were respiratory doctors (Table 1). Results from Australia and New Zealand were combined, given the smaller workforce and thus limited number of participants from New Zealand (21 palliative medicine specialists and 25 respiratory physicians).

**Table 1:** Participant demographics

	Palliative Medicine (*n* = 263)			Respiratory Medicine	Specialties Compared
	ANZ (*n* = 129)	UK (*n* = 134)	All (n = 263)	ANZ (*n* = 177)	
Female	80 (62.0%)	112 (83.6%)	192 (73.0%)	61 (34.4%)	*p* < 0.0001
Age					
25–35	25 (19.3%)	31 (23.1%)	56 (21.3%)	40 (22.6%)	*p* = 0.953
36–45	27 (20.9%)	50 (37.3%)	77 (29.3%)	57 (32.2%)	
46–55	31 (24.0%)	36 (26.9%)	67 (25.5%)	39 (22.0%)	
56–65	29 (22.5%)	15 (11.2%)	44 (16.7%)	27 (15.3%)	
> 65	11 (8.5%)	0	11 (4.2%)	8 (4.5%)	
Missing	6 (4.7%)	2 (1.5)	8 (3.0%)	6 (3.4%)	
Position					
Consultant	89 (69.0%)	107 (79.9%)	196 (74.5%)	145 (81.9%)	*p* = 0.068
Specialist trainee	40 (31.0%)	27 (20.1%)	67 (25.5%)	32 (18.1%)	
Mean years in specialty ^a^	12.5 (9.1)	12.3 (7.3)	12.4 (8.2)	15.0 (10.8)	*p* = 0.006
Mean no. of COPD patients seen/month ^a^	3.1 (3.1)	2.5 (3.4)	2.8 (3.3)	14.1 (12.0)	*p* < 0.0001

Data are represented as either frequencies or means (denoted by ^a^), with either proportions or standard deviations in parentheses

The majority of respiratory doctors (140, 80.9%) reported being very or quite comfortable providing a palliative approach to people with COPD. Only eight (4.6%) respiratory participants were quite or very uncomfortable offering this care, and the remainder were neutral. Respiratory doctors’ demographic characteristics and experience (including age, gender, country, specialty, position and location of practice, mean years worked in specialty and mean number of patients with severe COPD seen per month) were not associated with level of comfort providing a palliative approach.

Almost two thirds of respiratory doctors (113, 63.8%) recommended referring the person in the case vignette to specialist palliative care, with 90 (50.8%) recommending referring for long-term specialist palliative care input and 23 (13.0%) for an opinion only. Thirteen (7.3%) participants were uncertain if they would refer and the remainder would not. Female respiratory doctors were twice as likely as their male colleagues to recommend referral to the specialist palliative care service (OR = 2.0, 95%CI = 1.0–3.9, *p* = 0.048). No other demographic characteristics or experience were associated with respiratory doctors recommending referral to specialist palliative care.

Respiratory doctors referred people with COPD for many reasons including for: psychosocial and spiritual care (105, 59.3%), carer support including future bereavement support (104, 58.5%), and end-of-life care (94, 53.1%) (Table 2). Of the 64 (36.2%) respiratory physicians who would not or were unsure if they would refer to specialist palliative care, 27 (15.3%) reported being able to provide a palliative approach themselves, 19 (10.7%) reported difficulty accessing palliative care in their location and 13 (7.3%) thought the person described in the case vignette was unlikely to die within the next 12 months.

**Table 2:** Respiratory doctors’ referrals to specialist palliative care

Reasons for referring COPD patients to palliative care	*N* = 113^a^
For holistic care including psychosocial and spiritual care	105 (59.3%)
For carer support including future bereavement support	104 (58.8%)
For future end-of-life care	94 (53.1%)
To access community palliative care	92 (52.0%)
To access respite care	78 (44.1%)
For management of symptoms including breathlessness	75 (42.4%)
For completion of advance care planning	63 (36.2%)
Reasons for not referring COPD patients to palliative care	*N* = 64^b^
I am capable of providing generalist palliative care myself	27 (15.3%)
Palliative care services are difficult to access in my location	19 (10.7%)
I do not think the patient might die in the next 12 months	13 (7.3%)
I do not want the patient to feel abandoned by me	7 (4.0%)
The patient does not require specialist palliative care yet	6 (3.4%)
I do not want to take away the patient’s hope	4 (2.3%)
Previously have not found palliative care teams helpful for COPD patients	3 (1.7%)

Data are represented as frequencies with proportions of the total group (n = 177) in parentheses

^a^Only participants who said they would refer to palliative care were directed to answer this question

^b^ Only participants who said they would NOT/were unsure regarding referral to palliative care were directed to answer

Nearly all doctors working in both respiratory (172, 97.2%) and palliative medicine (260, 98.9%) reported that they would initiate a discussion with the person in the case vignette (or similar people) regarding prognosis and advance care planning. Only 4 doctors (all respiratory) reported that they would not initiate this discussion with the person in the case vignette (or similar patients), with 2 reporting lack of time as a barrier. Holding the belief that advance care planning should be undertaken was not associated with any of the participants’ demographic characteristics or experience for each specialty.

Both specialist groups recommended discussing multiple topics during ACP conversations including: mechanical ventilation and/or intensive care unit admission in the future, the utility of cardiopulmonary resuscitation, and the nature and role of palliative care (Table 3). Palliative medicine doctors were significantly more likely to recommend discussing: what palliative care is and whether indicated (*p* < 0.0001), what death and dying might be like from COPD (p < 0.0001), the likely prognosis (*p* = 0.004) and changing treatment goals to aim for palliation (*p* = 0.028).

**Table 3:** Recommended ACP discussion topics

	Resp Med	Pall Med
ACP topics		
The utility of CPR in the future	159 (89.8%)	249 (94.7%)
Mechanical ventilation and/or ICU admission in the future	164 (92.7%)	241 (91.6%)
What palliative care is and if this is indicated	139 (78.5%)	246 (93.5%)
What death and dying might be like from COPD	97 (54.8%)	200 (76.0%)
The likely prognosis in months and years	100 (56.5%)	191 (72.6%)
Changing treatment goals to be palliative	111 (62.7%)	191 (72.6%)
Appointing a medical power of attorney or substitute decision maker	123 (69.5%)	185 (70.3%)
Other treatment limitations	4 (2.3%)	20 (7.6%)
Other suggested topics		
Desired place of future care (home/hospital/hospice)	0	39 (14.8%)
Desired place of death	0	37 (14.1%)
The patient’s goals, values, wishes, hopes & fears	2 (1.1%)	27 (10.3%)
Ascertain the patient’s understanding and knowledge first	3 (1.7%)	4 (1.5%)
Patient led discussion	0	22 (8.4%)
Future wishes regarding antibiotics	3 (1.7%)	10 (3.8%)
Future wishes regarding NIV	3 (1.7%)	9 (3.4%)
Advance treatment directives	0	12 (4.6%)
Making a will or arranging affairs	1 (0.6%	7 (2.7%)

Data are represented as frequencies with proportions of the total group (n = 177) in parentheses

*CPR* Cardiopulmonary resuscitation, *ICU* Intensive Care Unit, *NIV* Non-invasive ventilation

Many themes were identified in the extensive free text comments participants wrote, including that specialists believed current palliative care arrangements for people with advanced COPD are inadequate and fragmented:

*“(This is a) difficult and big problem, and an area that clearly requires greater investment into formal palliative care services for this patient group, and greater formal systems of collaboration between respiratory physicians, palliative care teams, and community health providers.”* [Respiratory Medicine Specialist number 13, ANZ]

Fear of palliative care by both clinicians and patients was also noted as an issue:

*“Of our palliative care patients I think they have the greatest number of unmet needs, but often they or their clinicians are fearful of what palliative care entails.”* [Palliative Medicine Specialist number 39, ANZ]

There was also concern that both people with COPD and their caregivers experience issues such as anxiety:

*“COPD is hard to palliate. Patients find the exacerbations distressing and panic inducing. This also contributes to carer anxiety.”* [Palliative Medicine Specialist number 62, UK]

Additionally, limited available support from palliative care services currently was highlighted as an issue.

*“Unfortunately our local palliative care service is under-resourced to support similar patients to this due to the frequent longevity of their needs. This is likely to be a growing problem in the future.”* [Respiratory Medicine Specialist number 71, ANZ]

Finally, both respiratory and palliative medicine physicians raised concerns regarding each other’s knowledge and practices.

*“Palliative care physicians manage this (COPD patients) very poorly.”* [Respiratory Medicine Specialist number 4, ANZ]

*“It’s end organ disease - we need to enable respiratory physicians to manage their patients in all phases of the illness, and use consultative services when necessary, rather than ‘handing over’ care to palliative care when goals of care are palliative.”* [Palliative Medicine Specialist number 57, UK]

## Discussion

Recent international surveys of respiratory doctors have examined referral practices to specialist palliative care in the context of organisation of current service models, availability and local health policies [21, 30, 31]. In this large, multi-national study spanning three countries, in addition to examining recognised barriers to referring to specialist palliative care, we also uniquely identified that the majority of respiratory doctors are comfortable providing a palliative approach, but also recognise the role of specialist palliative care for people with advanced COPD. Additionally, both respiratory and palliative medicine doctors acknowledge the importance of ACP discussions and identify similar key topics to discuss. However, current working models are considered inadequate with insufficient communication and collaboration between the many health professionals often involved in caring for people with advanced COPD.

### Attitudes to palliative care

The vast majority of respiratory doctors from Australia and New Zealand, including doctors in specialist training, reported being very or quite comfortable providing a palliative approach to people with COPD. This finding is novel and may partly explain why people with COPD are infrequently referred to specialist palliative care services in Australia [15, 17]. Interestingly few participants in this study reported poor access to specialist palliative care services as an obstacle to referring COPD patients. By contrast respiratory physicians in the UK, Netherlands, and Sweden have cited lack of access to specialist palliative care services as an important barrier for people with COPD [32]. Instead, in this study participants highlighted that whilst specialist palliative care services were available, these services did not have the capacity or experience to manage advanced COPD patients, and importantly established relationships between respiratory and palliative medicine were absent.

Senior doctors’ attitudes to specialist palliative care have been described as a barrier to referring people with cancer to these services [33]. However, the majority of respiratory doctors in our study recognised the benefits of referring people with advanced COPD to specialist palliative care. However, some respiratory doctors focussed on the prognosis and limited survival, as opposed to patients’ needs when considering referral to specialist palliative care. Notably female doctors were considerably more likely to recommend referral to specialist palliative care. Likewise, when Australian junior doctors within their first five years of qualifying were surveyed (using this same questionnaire) regarding their knowledge and experiences managing people with advanced COPD, female trainees were approximately three times more likely to suggest referring to palliative care than male trainees [34]. This consistent finding requires further investigation to understand the reasons behind this difference in female doctors’ reported practices.

The principle reasons for referring to specialist palliative care in this survey were for access to psychosocial and spiritual care, carer support including bereavement support, and end-of-life care. A survey of British respiratory physicians also identified that the main reasons for seeking specialist palliative care for people with chronic lung diseases were for end-of-life care (63%) and psychological support (70%) [21]. Likewise, Australian junior doctors reported referring people with COPD to specialist palliative care to access community palliative care input (54%) and end-of-life care (45%) [34]. However, in the current study, respiratory doctors less commonly recommended referral for symptom management (42%), compared to 63% of British respiratory physicians and 62% of Australian junior doctors who referred for this reason [21, 34]. This would suggest that the ANZ respiratory doctors surveyed in this study not only feel comfortable providing a palliative approach, but also have greater confidence in managing difficult symptoms such as chronic breathlessness. However, there remains a significant need for specialist palliative care for people with COPD, particularly for psychosocial and existential support, addressing communication needs and for bereavement support of carers [11].

### Advance care planning

The significant need for good communication and ACP in people with COPD and their carers is well-described [35, 36], and in this study was almost universally recognised. The responsibility for ACP rests with respiratory doctors as suggested by the majority of respondents in this study. Notably, while both specialties suggested similar topics should be covered within ACP and that it may involve several discussions, palliative medicine doctors were more likely to address challenging topics such as death and dying, prognosis, treatment limitations and place of future care as part of these discussions. Similarly, palliative medicine doctors recommended patient-led ACP discussions. Both specialties therefore report being engaged with ACP and their different approaches and skills appear to be complementary over the series of ACP conversations a patient may require. Therefore ideally respiratory doctors should initiate these discussions, and palliative medicine doctors can then develop and expand the conversation as the illness progresses and according to each person’s needs and wishes.

### Models of care

Confidence providing a palliative approach, managing difficult symptoms such as chronic breathlessness and awareness regarding the importance of ACP in people with advanced COPD does not necessarily translate into practice. A number of authors have documented the significant unmet palliative care needs reported by COPD patients [35] and the infrequent engagement in ACP discussions [21, 22, 25, 35, 37], suggesting a mismatch between our participants’ responses and actual day-to-day clinical practice. Therefore it is not lack of awareness or confidence that is preventing clinicians from addressing the significant needs of people with advanced COPD. While lack of time may be a barrier, participants in this study raised concerns regarding the educational needs of doctors in each specialty and highlighted that current models of care are fragmented, with little collaboration between respiratory medicine, palliative medicine or primary care. Accessible, integrated, multi-disciplinary services, which embrace collaboration, trust and bi-directional education and support between both respiratory medicine and palliative care may overcome some of these issues [38]. Such integrated services include the Melbourne Advanced Lung Disease Service [39], and the London Breathlessness Support Service [40], both of which have demonstrated improved outcomes for people with COPD. However, larger, multi-site trials are required to fully assess these new models of care.

This study has some limitations. We had intended to survey British respiratory physicians, however, the British Thoracic Society declines to disseminate research surveys to its members. The response rate in this study, whilst similar to other online surveys of physicians [41–43], was low, thus limiting the generalisability of our findings. However, the gender and age characteristics of participants in this study were representative of the workforce demographics in all three countries [14, 17, 44–46]. Additionally, surveys require respondents to make black and white decisions to hypothetical scenarios, whereas in clinical practice these issues are not absolutely clear, instead requiring careful consideration.

## Conclusions

Both respiratory and palliative medicine doctors recognised the significant palliative care and ACP needs of people with advanced COPD. Respiratory doctors reported being comfortable providing a palliative approach and acknowledged the role of both specialist palliative care and ACP, yet in reality people with advanced COPD rarely access these elements of care. Additionally, current models of care for people with COPD were considered inadequate and fragmented. Collaboration, trust, and bi-directional education between respiratory and palliative medicine, perhaps through integrated, multidisciplinary services, are urgently required to address the unmet needs of people with advanced COPD.

## Abbreviations

ACP: Advance care planning
ANZ: Australia and New Zealand
COPD: Chronic obstructive pulmonary disease
